# Determinants of renal flow reserve in adult patients with and without renal artery stenosis

**DOI:** 10.14814/phy2.70572

**Published:** 2025-09-21

**Authors:** T. A. Bouwmeester, L. van de Velde, D. Collard, R. Delewi, A. B. G. N. Lamers, M. A. M. Beijk, R. J. de Winter, I. A. J. Zijlstra, L. Vogt, B. J. H. van den Born

**Affiliations:** ^1^ Department Vascular Medicine, Amsterdam Cardiovascular Sciences Amsterdam UMC, University of Amsterdam Amsterdam The Netherlands; ^2^ Department Interventional Cardiology Amsterdam UMC, University of Amsterdam Amsterdam The Netherlands; ^3^ Department Interventional Radiology Amsterdam UMC, University of Amsterdam Amsterdam The Netherlands; ^4^ Department Internal Medicine, Section Nephrology Amsterdam UMC, University of Amsterdam Amsterdam The Netherlands; ^5^ Amsterdam Cardiovascular Sciences Amsterdam UMC Amsterdam The Netherlands

**Keywords:** arteriolar resistance, dopamine, hyperemia, renal artery stenosis, renal blood flow, renal function

## Abstract

Renal flow reserve (RFR) is a hemodynamic measure of renal microvascular function and may help identify patients with renal artery stenosis (RAS) who could benefit from revascularization. Reference ranges and clinical correlates of RFR in patients with and without RAS remain unknown. We analyzed intra‐arterial renal flow velocity measurements from 76 participants with and without RAS, all with eGFR ≥30 mL/min/1.73m^2^. Each underwent baseline and dopamine‐induced hyperemic flow assessments using an intrarenal bolus of 30 μg/kg. RFR was defined as the ratio of mean hyperemic to baseline flow. Group differences were assessed using descriptive statistics and linear regression models adjusting for potential confounders. Median RFR was similar between participants with RAS (1.73, IQR 1.31–2.11) and those without (1.88, IQR 1.60–2.47). Lower RFR was associated with lower eGFR (*p* = 0.027). Higher RFR values were observed in participants using beta‐blockers (*p* = 0.010), independent of age, sex, eGFR, and blood pressure. RFR was negatively associated with eGFR and positively with beta‐blocker use. No associations were found with age, sex, hypertension, diabetes, or presence of RAS. The link with beta‐blockers may be caused by interactions with the systemic and renal dopaminergic system.

## 1 INTRODUCTION

Renal artery stenosis (RAS) is a common cause of secondary hypertension, often leading to treatment resistance and progressive kidney function decline (Weber & Dieter, 2014). Optimal drug therapy forms the cornerstone for the treatment of RAS, but selected patients may benefit from invasive interventions such as percutaneous transluminal angioplasty (PTA) with or without stenting (Hicks et al., 2022). At present, it remains difficult to predict which patients will benefit from PTA as angiographic stenosis grading correlates only modestly with the hemodynamic consequences of the stenosis (Subramanian et al., 2005), while multiple randomized controlled trials have failed to show an additional therapeutic effect of PTA on top of optimal medical therapy (ASTRAL Investigators et al., 2009; Bax et al., 2009; Cooper et al., 2014).

Intra‐arterial hemodynamic measurements may help to identify patients who have a higher probability of a beneficial treatment response after stenting or balloon angioplasty (Gomes Junior et al., 2018; Subramanian et al., 2005; van Brussel et al., 2017). Hemodynamic measurements usually consist of pressure measurements, flow (velocity) measurements, or a combination. Commonly used parameters include the translesional pressure gradient and the renal fractional flow reserve (rFFR), which is defined as the ratio of the post‐stenotic to pre‐stenotic pressure during induced hyperemia. Whereas rFFR is predominantly a measure of the hemodynamic effect of the stenosis, renal flow reserve (RFR) provides insight into the capacity of the renal microvasculature to increase flow rate. The combined measurement could therefore identify stenoses that are hemodynamically significant (low rFFR) and have exceeded the kidney's regulatory capacity (low RFR), indicating an expected benefit from PTA (van Brussel et al., 2017).

Besides a possible tool in selecting patients with RAS, the quantification of nephron function through RFR and the related renal functional reserve may also help to improve our understanding of chronic kidney disease (Barai et al., 2010) and acute kidney injury (Husain‐Syed et al., 2019; Ullah et al., 2024). Earlier studies have primarily focused on the ability to increase glomerular filtration rate, but performing an invasive intervention allows us to uniquely measure the potential increase in renal blood flow in response to dopamine stimulation. Before we are able to use RFR as a diagnostic tool, normal values of RFR in a reference population are required.

Whether RFR is decreased in patients with RAS and how it relates to other determinants of renal blood flow remains to be clarified. We therefore assessed whether intra‐arterial flow measurements in participants with and without RAS were associated with clinical characteristics known to influence renal blood flow.

## 2 MATERIALS AND METHODS

### 2.1 Patient inclusion

We included 76 participants of the HEmodynamics in Renal Artery stenosis (HERA) studies. The HERA studies were designed to assess the feasibility and reproducibility of intra‐arterial hemodynamic measurements in patients with and without RAS (HERA‐1) (van Brussel et al., 2020) maximal and minimal values of renal flow in healthy subjects (HERA‐2) (Collard et al., 2020, 2023), and to assess the diagnostic value of intra‐arterial pressure‐flow measurements in patients with RAS (HERA‐3). Participants were included from March 2014 to March 2024.

The HERA‐1 study included participants scheduled for an elective coronary or renal angiography, both with and without renal artery stenosis. The HERA‐2 study included participants scheduled for either a coronary‐, renal‐, or peripheral angiography, both with and without renal artery stenosis. The HERA‐3 study only included participants scheduled for a renal angiography, who were suspected or confirmed to have a renal artery stenosis. In a subset of participants, CT angiography was used to manually segment the kidneys to obtain cortex and parenchymal volumes of the kidney at the side where the measurement was performed. Common inclusion criteria were clinically stable patients aged over 18 years. Participants with severe heart failure (NYHA class > II) were excluded to ensure that only clinically stable patients were included in the study. Moreover, systemic dopamine was administered during the study, which carries additional risks for individuals with severe heart failure. Additional exclusion criteria included women of childbearing age not on active birth control, known and active atrial fibrillation, the inability to sign an informed consent, or an estimated glomerular filtration rate (eGFR) < 30 mL/min/1.73m^2^. In HERA‐1, patients over the age of 75 were excluded. For this specific analysis of RFR, we further excluded patients with a flow measurement of insufficient quality as assessed by two independent investigators.

The study was conducted in accordance with the Declaration of Helsinki and approved by the local ethics committee. The study was registered at the Dutch national trial registry (toetsing online, NL40795.018.12, NL‐OMON47085, NL‐OMON48149), and all participants provided written informed consent.

### 2.2 Patient characteristics

Participants attended a baseline visit where their age, ethnicity, height, weight, office systolic and diastolic blood pressure, medical history, medication use, and history of renal artery interventions were recorded. RAS was assessed during renal angiography and defined by the performing interventional radiologist as a visually significant stenosis, based on the angiographical images. Hypertension was defined as the use of antihypertensive medication or an office blood pressure ≥140/90 mmHg at baseline. We defined a history of antihypertensive medication use as the use of ACE inhibitors, angiotensin‐receptor blockers, calcium‐channel blockers, or diuretics. Participants using only beta‐blockers, without any other antihypertensive agents, were not classified as having a history of antihypertensive use. A history of diabetes was defined as self‐reported, and a morning urine sample was taken for the assessment of albuminuria on the day of the angiography. Increased albuminuria was defined as >3 mg/mmol in a collected urine sample.

### 2.3 Hemodynamic measurements

All measurements were performed by experienced interventional radiologists or cardiologists, with the study team present in the intervention room. Intra‐arterial pressure‐flow measurements were performed using a 0.014″ Combowire (Philips‐Volcano, San Diego, CA), which at its tip is equipped with a pressure sensor and a Doppler ultrasound sensor. Pressure and flow velocity signals were stored digitally at a frequency of 200 Hz. During the study procedures, the tip of the wire was positioned at least three reference diameters distally from the stenosis to account for post‐stenotic flow disturbances. Measurements were taken after a stable Doppler flow velocity signal was acquired, guided by audio and visual feedback. Aortic or proximal renal artery pressure was measured using an external transducer (Namic, Navilyst Medical, Marlborough, MA) attached to the continuously flushed guiding sheath, positioned either at the origin of the artery if an ostial stenosis was present, or in the renal artery, proximal to the stenosis. Hyperemia was induced with an intrarenal bolus of 30 μg/kg dopamine over 60 s. A subset of participants was subjected to a static handgrip procedure in order to induce minimal renal blood flow.

Custom software written in MATLAB (R2018b; The Mathworks, Inc.) was used to analyze the data. A stable segment of at least three, and maximum 15 consecutive beats was chosen to calculate flow at either baseline, or hyperemia.

RFR was defined as the ratio between hyperemic flow velocity and baseline flow velocity, as earlier research has shown the diameter of the renal artery remains constant during the induction of hyperemia using a dopamine bolus (Manoharan et al., 2006). rFFR was defined as the ratio between mean distal pressure and mean aortic pressure during hyperemia.

### 2.4 Follow‐up measurements

In the HERA‐3 participants, we performed PTA, with or without stenting, if an angiographically significant stenosis was present. Patients attended a follow‐up meeting after 3 months to assess any change in blood pressure or the use of blood pressure‐lowering medication. Participants of the HERA‐3 who did not receive PTA were also asked to attend the follow‐up visit as a reference group. A successful intervention was defined as a ≥10 mmHg reduction in systolic blood pressure or a reduction in antihypertensive medication use.

### 2.5 Statistical analyses

Patient characteristics are described as mean (SD) and median (IQR) based on the distribution of the data. Differences in patient characteristics between groups were calculated using Kruskal–Wallis tests, ANOVA tests, or chi‐squared tests, based on data type and distribution.

Differences in unadjusted values of RFR between groups were calculated using the Wilcoxon rank test as the values of RFR were non‐normally distributed. Adjusting for possible confounding was performed using linear regression models. All significance levels were set for a two‐sided comparison at a *p* value < 0.05.

We used the subset of participants from the HERA‐3 study who underwent both a successful hemodynamic measurement and a baseline and follow‐up study visit to perform an additional exploratory analysis. In this analysis, we visualized trends between intervention success and renal hemodynamics by creating a basic plot with rFFR on the x‐axis and RFR on the y‐axis.

## 3 RESULTS

### 3.1 Baseline characteristics

**FIGURE 1:**
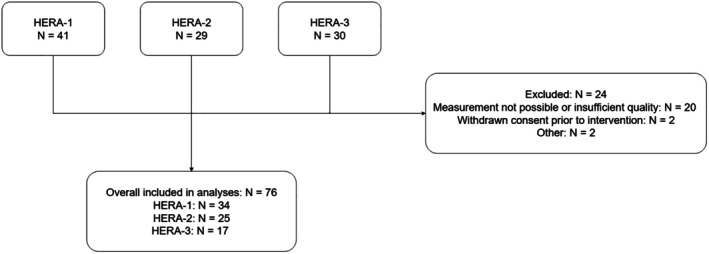
Flowchart of inclusion of the HERA1, HERA2, and HERA3 participants.

A total of 100 participants were included in the three studies combined, of whom 76 had received a flow measurement of sufficient quality for further analysis (Figure 1). The baseline characteristics of the 76 participants included for the present analysis are shown in Table 1. Overall, we included 49 male and 27 female participants, with a median age of 57 (IQR 50–67) years, 89% of European origin. Systolic BP was comparable between the three studies, with median values ranging from 139 [IQR 125–153] mmHg in the HERA‐2 study to 144 [IQR 129–154] mmHg in the HERA‐1 study. All participants from the HERA‐3 study used antihypertensive medication, while 60% of the participants in the HERA‐2 study and 77% of the participants in the HERA‐1 study used antihypertensive medication. Median eGFR was 89.6 [IQR 70.5–100.1] mL/min/1.73m^2^ in HERA‐1, 89.0 [IQR 78.8–99.2] mL/min/1.73m^2^ in HERA‐2, and 80.1 [IQR 48.2–93.6] mL/min/1.73m^2^ in HERA‐3 participants. Overall, 17 participants had a significant RAS as determined by the interventional radiologist and on‐site researcher based on the angiographical images.

**TABLE 1:** Baseline table of included participants.

	Overall	Hera 1	Hera 2	Hera 3	*p*
*N*	76	34	25	17	
Sex = Male (%)	49 (64.5)	23 (67.6)	16 (64.0)	10 (58.8)	0.823
Age (median [IQR])	57.0 [49.8, 67.0]	58.0 [52.0, 64.8]	58.0 [42.0, 68.0]	53.0 [49.0, 64.0]	0.823
Ethnicity = European descent (%)	68 (89.5)	32 (94.1)	23 (92.0)	13 (76.5)	0.135
BMI (mean (SD))	27.1 (3.8)	27.1 (4.3)	27.6 (3.1)	26.5 (3.9)	0.706
Active smoking (%)					0.013
No	45 (59.2)	15 (44.1)	19 (76.0)	11 (64.7)	–
Unknown	3 (3.9)	0 (0.0)	1 (4.0)	2 (11.8)	–
Yes	28 (36.8)	19 (55.9)	5 (20.0)	4 (23.5)	–
Systolic BP (median [IQR])	141 [130, 154]	144 [129, 154]	139 [125, 153]	142 [135, 155]	0.362
Diastolic BP (median [IQR])	83 [74, 91]	74 [69, 89]	84.5 [79, 89]	90 [84, 96]	0.009
Hypertension present (%)	69 (90.8)	30 (88.2)	22 (88.0)	17 (100.0)	0.329
DM2 = Yes (%)	15 (19.7)	11 (32.4)	3 (12.0)	1 (5.9)	0.040
History of CVD = Yes (%)	44 (57.9)	23 (67.6)	11 (44.0)	10 (58.8)	0.191
Number of antihypertensives					0.250
0	9 (11.8)	6 (17.6)	3 (12.0)	0 (0.0)	–
1	14 (18.4)	4 (11.8)	7 (28.0)	3 (17.6)	–
2	27 (35.5)	13 (38.2)	9 (36.0)	5 (29.4)	–
3+	26 (34.2)	11 (32.4)	6 (24.0)	9 (52.9)	–
Alpha1blocker (*n* (%))	5 (6.6)	2 (5.9)	0 (0.0)	3 (17.6)	0.075
Beta‐blocker (*n* (%))	47 (61.8)	21 (61.8)	18 (72.0)	8 (47.1)	0.263
Calcium antagonist (*n* (%))	39 (51.3)	15 (44.1)	13 (52.0)	11 (64.7)	0.381
ACE‐blocker or ARB (*n* (%))	43 (56.6)	21 (61.8)	9 (36.0)	13 (76.5)	0.024
Diuretic (*n* (%))	22 (28.9)	10 (29.4)	4 (16.0)	8 (47.1)	0.093
Creatinine (median [IQR])	82.0 [75.5, 97.2]	87.5 [76.0, 97.8]	81.0 [78.0, 86.0]	85.0 [74.0, 127.0]	0.282
eGFR (median [IQR])	88.2 [70.0, 99.5]	89.6 [70.5, 100.1]	89.0 [78.8, 99.2]	80.1 [48.2, 93.6]	0.218
Albumin/creatinin	2.0 [0.8, 8.8]	2.1 [0.5, 6.6]	1.4 [0.8, 2.7]	4.5 [1.2, 15.8]	0.331
Albuminuria classification (%)					0.592
A1	28 (60.9)	12 (60.0)	8 (80.0)	8 (50.0)	–
A2	12 (26.1)	6 (30.0)	1 (10.0)	5 (31.2)	–
A3	6 (13.0)	2 (10.0)	1 (10.0)	3 (18.8)	–
Albuminuria >3 mg/mmol	18 (39.1)	8 (40.0)	2 (20.0)	8 (50.0)	0.311
Indication for intervention (%)					<0.001
Cardiac	38 (50.0)	22 (64.7)	16 (64.0)	0 (0.0)	–
Renal	35 (46.1)	11 (32.4)	7 (28.0)	17 (100.0)	–
Peripheral artery disease	2 (2.6)	0 (0.0)	2 (8.0)	0 (0.0)	–
Cardiac and renal	1 (1.3)	1 (2.9)	0 (0.0)	0 (0.0)	–
Renal artery stenosis (%)	16 (21.1)	4 (11.8)	1 (4.0)	11 (64.7)	<0.001
Measurement left side (%)	31 (40.8)	14 (41.2)	10 (40.0)	7 (41.2)	0.995

Abbreviations: ACE, angiontensin converting enzyme; ARB, angiotensin receptor blocker; BMI, body mass index; BP, blood pressure; CVD, cardiovascular disease; DM, diabetes mellitus; eGFR, estimated glomerular filtration rate; IQR, interquartile range.

**FIGURE 2:**
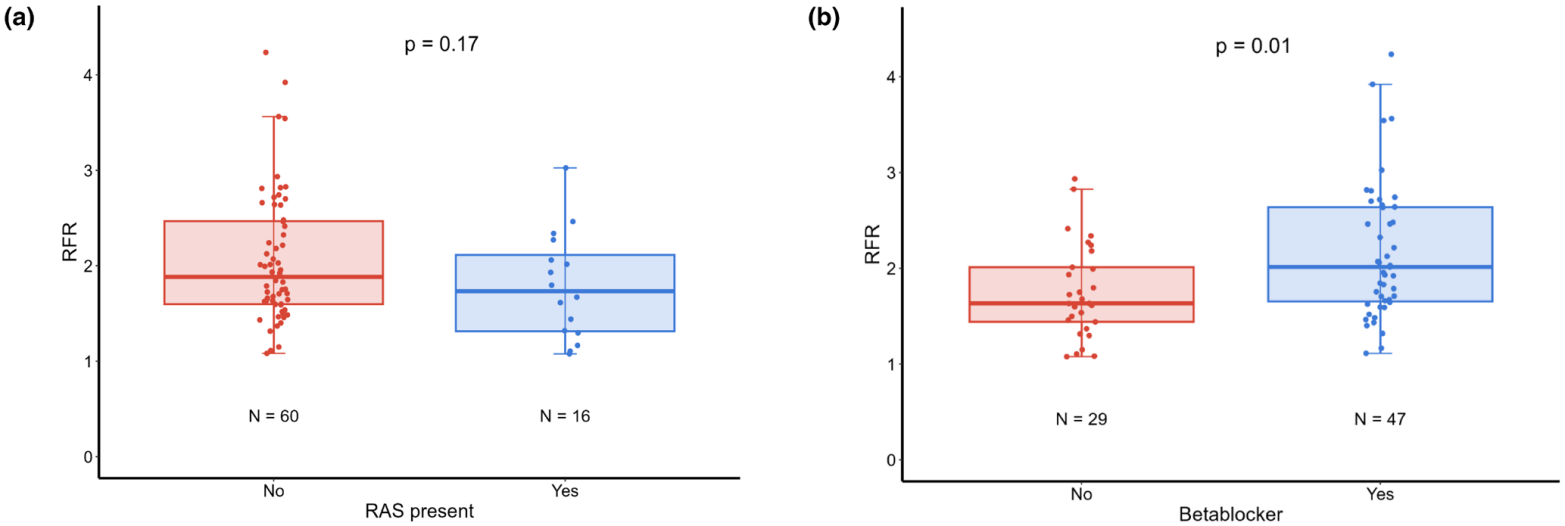
(a and b) Renal flow reserve in participants with and without renal artery stenosis (RAS) and present use of beta‐blocking medication. RAS, renal artery stenosis; RFR, renal flow reserve. RAS was defined as the presence of a visually significant stenosis based on the angiographical images obtained during the intervention.

RFR ranged from 1.08 – 4.20, with a median value of 1.84 (IQR 1.58–2.36) in the total cohort. There was no significant difference in RFR between the participants of the different HERA studies. Median RFR was 1.73 (IQR 1.31–2.11) in participants with RAS, and 1.88 (1.60–2.47) in participants without RAS (*p* = 0.17, Figure 2a).

Table 2 shows the differences in RFR between participant categories. There were no differences in median RFR between male and female participants (*p* = 0.46) or between participants of European descent and those of non‐European descent (*p* = 0.24). Additionally, we did not find a difference in RFR between participants with and without type 2 diabetes (*p* = 0.78) or between those with and without hypertension (*p* = 0.84). Participants using beta‐blockers had a significantly higher median RFR (2.01 [IQR 1.65–2.64]) than those not on beta‐blocking medication (1.63 [IQR 1.44–2.01], *p* = 0.01) (Figure 2b), while no significant association between RFR and other types of antihypertensive agents was detected. After adjusting for age and sex in the regression models (Table 3), the significant difference in RFR between participants with and without beta‐blocking agents persisted. Similarly, additional adjustment for systolic blood pressure, eGFR, and the presence of RAS had no material effect on the association.

**TABLE 2:** RFR values per participant category.

	*N*	RFR, median [IQR]	*p*
Sex			
Men	49	1.80 [1.60, 2.64]	0.464
Women	27	1.85 [1.52, 2.02]	
Ethnicity			
European	68	1.93 [1.58, 2.46]	0.236
Other	8	1.63 [1.56, 1.88]	
DM‐2			
DM‐2	15	1.75 [1.64, 2.47]	0.779
No DM‐2	61	1.93 [1.52, 2.34]	
RAS			
RAS present	16	1.73 [1.31–2.11]	0.173
No RAS present	60	1.88 [1.60–2.47]	
Hypertension			
Hypertension	69	1.85 [1.54, 2.34]	0.839
No hypertension	7	1.66 [1.64, 2.25]	
Antihypertensive medication			
Uses antihypertensive medication	58	1.88 [1.54, 2.33]	0.798
No antihypertensive medication	18	1.80 [1.63, 2.42]	
Increased albuminuria			
Increased albuminuria	18	1.95 [1.68, 2.18]	0.804
No increased albuminuria	28	1.81 [1.59, 2.26]	
Alpha‐1‐blockers			
Uses alpha‐1‐blockers	5	1.93 [1.17, 2.07]	0.483
No alpha‐1‐blockers	71	1.83 [1.59, 2.38]	
Beta‐blockers			
Uses beta‐blockers	47	2.01 [1.65, 2.64]	0.010
No beta‐blockers	29	1.63 [1.44, 2.01]	
Calcium channel blockers			
Uses calcium channel blockers	39	1.71 [1.45, 2.33]	0.226
No calcium channel blockers	37	1.93 [1.65, 2.48]	
ACE‐inhibitors and/or ATR blockers			
Uses ACE‐inhibitors and/or ATR blockers	43	1.93 [1.60, 2.44]	0.460
No ACE‐inhibitors and/or ATR blockers	33	1.75 [1.54, 2.24]	
Diuretics			
Uses diuretics	22	1.73 [1.46, 2.10]	0.247
No diuretics	54	1.94 [1.60, 2.48]	

Abbreviations: ACE, angiotensin‐converting enzyme; ATR, angiotensin II receptor; DM‐2, Type 2 diabetes mellitus; IQR, interquartile range, RAS, renal artery stenosis.

**TABLE 3:** Regression coefficients of RFR and continuous determinants.

Variable	Model 1			Model 2		
	β	95% CI	*p* Value	β	95% CI	*p* Value
Age (per 10 years)	−0.05	−0.16 to 0.06	0.366			
Sex—Male	0.13	−0.19 to 0.44	0.420			
Ethnicity—European	0.29	−0.20 to 0.78	0.242	0.39	−0.12 to 0.90	0.133
BMI (per 10 kg/m^2^)	0.11	−0.29 to 0.51	0.580	0.12	−0.29 to 0.52	0.561
Diabetes present	0.05	−0.33 to 0.43	0.783	0.03	−0.36 to 0.42	0.880
RAS present	−0.27	−0.64 to 0.09	0.140	−0.27	−0.64 to 0.10	0.153
eGFR (per 10 mL/min)	0.07	0.01 to 0.14	0.027	0.08	−0.01 to 0.16	0.072
Alb/Creatinin ratio (per 10 increase)	−0.03	−0.06 to 0.01	0.137	−0.02	−0.06 to 0.01	0.223
Alb/Creatinin ratio (log transformed)	−0.05	−0.14 to 0.05	0.331	−0.04	−0.14 to 0.06	0.454
Increased albuminuria	−0.01	−0.35 to 0.34	0.972	0.01	−0.34 to 0.36	0.946
Hypertension present	−0.18	−0.70 to 0.35	0.506	−0.16	−0.68 to 0.37	0.556
Use of antihypertensive medication	−0.07	−0.43 to 0.29	0.692	−0.09	−0.45 to 0.27	0.622
Systolic BP (per 10 mmHg)	−0.05	−0.13 to 0.03	0.199	−0.04	−0.12 to 0.04	0.292
Diastolic BP (per 10 mmHg)	0.01	−0.11 to 0.13	0.904	−0.01	−0.14 to 0.13	0.936
Mean arterial pressure (per 10 mmHg)	−0.03	−0.15 to 0.08	0.578	−0.01	−0.14 to 0.13	0.936
Pulse pressure (per 10 mmHg)	−0.07	−0.16 to 0.02	0.107	−0.07	−0.17 to 0.03	0.187
Parenchymal volume (per 10 cm^3^)	0.03	−0.02 to 0.07	0.243	0.03	−0.04 to 0.1	0.341
Use of alpha‐1‐blocker	−0.28	−0.89 to 0.33	0.362	−0.27	−0.88 to 0.34	0.379
Use of beta‐blockers	0.40	0.10 to 0.70	0.009	0.51	0.20 to 0.82	0.002
Use of calcium channel blockers	−0.10	−0.41 to 0.20	0.493	−0.15	−0.46 to 0.16	0.339
Use of ACE‐blocker/ARB	0.12	−0.18 to 0.42	0.435	0.12	−0.19 to 0.43	0.430
Use of diuretics	−0.24	−0.57 to 0.09	0.148	−0.25	−0.58 to 0.08	0.130
Cortex volume (per 10 cm^3^)	0.04	−0.02 to 0.1	0.223	0.04	−0.04 to 0.13	0.297
rFFR (per 0.1 increase)	−0.004	−0.14 to 0.13	0.952	−0.013	−0.15 to 0.13	0.856

*Note*: Model 1 is a univariate model (unadjusted), model 2 is adjusted for age and sex. Parenchymal and cortex volumes have been assessed in a subset of participants (*n* = 17).

Abbreviations: ACE, angiotensin converting enzyme; alb, albumin; ARB, angiontensin receptor blocker; BMI, body mass index; BP, blood pressure; eGRF, estimated glomerular filtration rate; RAS, renal artery stenosis; rFFR, renal fractional flow reserve.

Table 3 shows the regression coefficients of the independent variables and their relation with RFR. There was a significant positive association between eGFR and RFR in the univariate model (*p* = 0.03), which was attenuated after adjusting for age and sex (*p* = 0.07), while no significant association between RFR and albuminuria was found. No association between RFR and age, BMI, BP, or kidney volume was found. We did not find an association between RFR and rFFR (*p* = 0.86).

**FIGURE 3:**
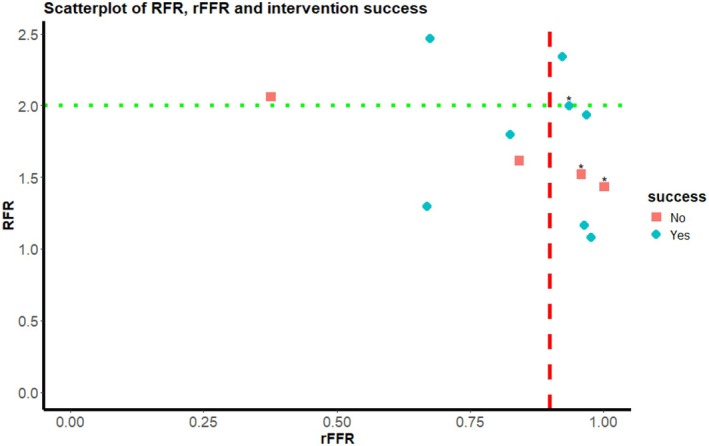
Scatter plot of rFFR, RFR, and intervention success. Asterixis depicts participants that did not undergo a PTA with or without stenting. A successful follow‐up period was defined as a ≥10 mmHg reduction in systolic blood pressure or a decrease in antihypertensive medication use. Values on both axes represent unitless ratios. rFFR, renal fractional flow reserve; RFR, renal flow reserve.

In the HERA‐3 study, 12 participants underwent a hemodynamic measurement and attended both the baseline and follow‐up visits. Of these, nine received an intervention due to a radiologically significant stenosis, while three did not, as their stenosis was not deemed significant based on angiographic imaging. Within this subset, we found no clear association between rFFR, RFR, and intervention success (Figure 3).

## 4 DISCUSSION

In the present study, we used invasive hemodynamic measurements to assess renal blood flow reserve in 76 patients with and without RAS. RFR in patients with RAS tended to be lower compared to those without RAS, and RFR was positively associated with eGFR and the use of beta‐blockers. The data provide a point of reference for RFR values in participants with normal to mildly decreased eGFR, supporting studies investigating the clinical utility of RFR.

The association between RFR and eGFR corresponds well to earlier research showing altered renal hemodynamics in patients with loss of kidney function (Barai et al., 2010; Bosch et al., 1986). Similarly, previous studies have shown a progressive loss in renal functional reserve, which measures excess filtration capacity rather than flow capacity, across CKD stages (Barai et al., 2010), as well as a predictive value for functional recovery after acute kidney injury (Ullah et al., 2024). In our group of non‐RAS patients, renal flow reserve (1.89) was markedly higher than renal functional reserve in a group of CKD stage 1 patients (1.19) (Barai et al., 2010), suggesting that RFR could be more sensitive to assess early changes in kidney function for pathologic kidney processes and its response to treatments. Glomerular hyperfiltration often occurs in the early stages of kidney disease and is characterized by high eGFR and low RFR, which might even blunt the association between eGFR and RFR found in our study (Palatini, 2012).

We found that participants using beta‐blockers had a higher RFR than those without. Although participants using beta‐blocking medications were significantly older than those without, adjusting for age and sex only increased the strength of this association. Further adjustments for eGFR, the presence of a renal artery stenosis and systolic blood pressure did not change these findings. This finding was unsuspected as we initially postulated that higher renal sympathetic nerve activity decreases baseline renal arterial blood flow through vasoconstriction (Drew, 2017; Momen et al., 2004, 2008), and would therefore be accompanied by higher RFR values, because of a lower baseline flow rate. Previous animal studies, however, have shown that beta‐1‐blocking agents diminish dopamine synthesis in the brain (Tuross & Patrick, 1986), and it is possible that this effect is also present in the renal dopamine system (Carey, 2001). Therefore, it is conceivable that a dopamine bolus in a dopamine‐depleted environment with subsequent upregulated dopamine receptors leads to an increased hemodynamic effect. This is further supported by an earlier study showing that both participants with an without beta‐blocking agents had a similar absolute increase in cardiac output upon receiving a dopamine infusion, however, the relative increase in cardiac output and in extension RFR was higher in those using beta‐blocking agents (Olsen et al., 1994).

We did not observe that participants with RAS had an impaired ability to increase blood flow to the affected kidney. However, our findings point in the same direction as those of an earlier study by Mounier‐Vehier et al. (2004), where RFR in stenosed renal arteries was compared with RFR in the contralateral normal renal artery from the same patient. Differences in RFR between normal and stenosed arteries in that study were higher compared to our findings, which could be explained by the higher stenosis grade of their included participants. In our combined three cohorts, we only included five participants with a renal FFR < 0.9, compared to 17/19 stenosis assessed as >70% stenosis in Mounier‐Vehier's study. Similarly, a study by Paivarinta et al. did not detect any differences in RFR between stenosed and healthy renal arteries. However, they indirectly assessed RBF from a single compartment model fitted to perfusion measurements and used enalapril to assess RFR (Paivarinta et al., 2018). Both factors limit the sensitivity of the method to detect changes in RFR.

Analogous to the coronary flow reserve (CFR), RFR can be interpreted as a quantification of the function of the renal microvascular bed. Therefore, we would expect RFR to be lower in patients with a history of diabetes or hypertension, which are known to damage the renal microvasculature (Chade, 2017), and that it would be positively correlated with kidney function. However, we did not observe lower RFR values in these patient groups.

In coronary hemodynamics, CFR has shown to improve the selection of patients that are likely to benefit from percutaneous coronary intervention, even on top of FFR measurements (Garcia et al., 2019). Notwithstanding the potential of rFFR as a method to assess the hemodynamic significance of RAS, as has been shown by earlier intervention studies, there is no clear diagnostic value in RFR measurements for improving patient selection for interventional treatment in patient with RAS (Gomes Junior et al., 2018; Subramanian et al., 2005; van Brussel et al., 2017).

An obvious limitation of our RFR assessment is its invasive nature, which prohibits its practical usefulness. The use of contrast‐enhanced ultrasound flow or perfusion assessment (Wang & Mohan, 2016) or MRI flow assessment (Villa et al., 2020), in combination with intravenous dopamine administration (Kishimoto et al., 2003), could be viable noninvasive alternatives. The use of intravenous dopamine reduces the measurement signal‐to‐noise ratio; however, as only about a quarter of flow increase was obtained for the intravenous route compared to the intra‐arterial renal route (Manoharan et al., 2006). Secondly, we excluded those with an eGFR <30 mL/min/1.73m^2^, which limits the generalizability of our findings to patients with CKD. Lastly, the relatively low number of participants with significant RAS on angiography limits both the statistical power and the generalizability of our findings.

## 5 CONCLUSION

In the present study, we measured RFR in 76 patients with and without RAS, and we show that RFR is significantly associated with eGFR and the use of beta‐blocking agents.

## AUTHOR CONTRIBUTIONS

T.A. Bouwmeester, L. van de Velde, R.J. de Winter, D. Collard, L. Vogt, and B.J.H. van den Born conceived and designed the research; T.A. Bouwmeester, L. van de Velde, D. Collard, R. Delewi, M.A.M. Beijk, IJ.A.J. Zijlstra, A.B.G.N. Lamers, and R.J. de Winter performed experiments; T.A. Bouwmeester and L. van de Velde interpreted the results of experiments; T.A. Bouwmeester prepared figures; T.A. Bouwmeester, L. van de Velde, D. Collard, and B.J.H. van den Born drafted the manuscript; all authors edited and revised the manuscript; all authors approved the final version of the manuscript.

## FUNDING INFORMATION

This work was funded, in part, by an innovation grant (Project No. 19OI18) from the Dutch Kidney Foundation and by the involved medical departments (Department of Internal Medicine, section Vascular Medicine; Amsterdam Cardiovascular Sciences; Amsterdam University Medical Centers).

## CONFLICT OF INTEREST STATEMENT

Philips provided in‐kind support for the Combowires, but had no role in the funding and design of this study. No conflicts of interest, financial or otherwise, are declared by the authors.

## ETHICS STATEMENT

The study was carried out in accordance with the Declaration of Helsinki, with all patients providing written informed consent before participation and following approval by the local medical research ethics committee.

## Supporting information


Table S1.

## Data Availability

The unaltered data with which the analyses of this study are performed is available on reasonable request.
