# Supplementary material for: Determinants of renal flow reserve in adult patients with and without renal artery stenosis
Source: Physiol Rep. 2025 Sep 21;13(18):e70572. doi: 10.14814/phy2.70572 (PMC12451016; doi:10.14814/phy2.70572)
Supplement: Supplementary file 1 — Table S1. [file PHY2-13-e70572-s001.docx]

**Supplemental material**

Tables: 1

**Supplemental Table 1**: Participant baseline characteristics, stratified by the us of beta-blocking medication.

|  | No Beta-blocker | Beta-blocker | p |
| --- | --- | --- | --- |
| N | 29 | 47 |  |
| Sex = Male (%) | 19 (65.5) | 30 ( 63.8) | 1.000 |
| Age (median [IQR]) | 53.0 [37.0, 60.0] | 61.0 [53.0, 68.0] | 0.011 |
| Ethnicity2 = Caucasian (%) | 22 (75.9) | 46 ( 97.9) | 0.008 |
| BMI (mean (SD)) | 27.2 (3.9) | 27.1 (3.8) | 0.857 |
| Smoking2 (%) |  |  | 0.810 |
| No | 16 (55.2) | 29 ( 61.7) | |
| Unknown | 1 ( 3.4) | 2 ( 4.3) |  |
| Yes | 12 (41.4) | 16 ( 34.0) | |
| Systolic BP (median [IQR]) | 142.5 [136.0, 151.0] | 140.0 [123.5, 154.5] | 0.285 |
| Diastolic BP (median [IQR]) | 86.0 [80.5, 92.0] | 79.0 [69.8, 89.5] | 0.052 |
| Hypertension present | 28 (96.6) | 41 ( 87.2) | 0.339 |
| Antihypertensive medication use | 21 (72.4) | 37 ( 78.7) | 0.726 |
| Alpha-1-blocker use = yes | 2 ( 6.9) | 3 ( 6.4) | 1.000 |
| Calcium channel blocker use = yes | 16 (55.2) | 23 ( 48.9) | 0.770 |
| ACE/ARB use = yes | 17 (58.6) | 26 ( 55.3) | 0.965 |
| Diuretic use = yes | 6 (20.7) | 16 ( 34.0) | 0.324 |
| DM-2 = yes | 5 (17.2) | 10 ( 21.3) | 0.894 |
| Creatinin (median [IQR]) | 83.5 [75.5, 100.5] | 82.0 [76.2, 96.2] | 0.694 |
| eGFR (median [IQR]) | 89.0 [75.3, 100.8] | 88.2 [69.2, 95.7] | 0.460 |
| Albumin/creatinine ratio (median [IQR]) | 2.1 [1.3, 9.4] | 2.0 [0.8, 7.4] | 0.558 |
| RAS present = yes | 8 (27.6) | 8 ( 17.0) | 0.419 |
| Parenchym volume (cm^3^) | 109.3 [83.3, 134.6] | 123.8 [106.9, 145.0] | 0.368 |
| Cortex volume (cm^3^) | 148.7 [119.6, 189.8] | 178.1 [154.7, 211.6] | 0.223 |

BMI = body mass index, BP = blood pressure, DM = diabetes mellitus, CVD = cardiovascular disease, ACE = angiontensin converting enzyme, ARB = angiotensin receptor blocker, eGFR = estimated glomerular filtration rate
